# Early Assessment of the Environmental Impact of Pentaspline Pulsed Field Ablation and Cryoablation in the Treatment of Paroxysmal Atrial Fibrillation

**DOI:** 10.36469/001c.151216

**Published:** 2025-12-15

**Authors:** Julian Chun, Boris Schmidt, Ines Timmermanns, Steffen Uffenorde, Carla Fernández-Barceló, Tobias Muench, Clare Brooke, Domenico Giovanni Della Rocca

**Affiliations:** 1 CCB – Medizinisches Versorgungszentrum Frankfurt und Main-Taunus GbR, Frankfurt, Germany; 2 Boston Scientific Medizintechnik GmbH, Düsseldorf, Germany; 3 Coreva Scientific GmbH & Co. KG, Koenigswinter, Germany; 4 Boston Scientific Limited, Hemel Hempstead, UK; 5 Heart Rhythm Management Centre, Postgraduate Program in Cardiac Electrophysiology and Pacing Universitair Ziekenhuis Brussels, Belgium

**Keywords:** cardiac ablation, sustainability, environmental impact, decision-analytic model, anesthesia, sedation

## Abstract

**Background:**

Atrial fibrillation (AF) affects 2% to 4.5% of the population. Catheter ablation, a key strategy for paroxysmal AF management, can be achieved through radiofrequency (RFA), cryoablation (CBA), or pulsed field ablation (PFA). While clinical outcomes are well studied, their environmental impact remains underexplored.

**Objectives:**

This study modeled the environmental impact of CBA and PFA ablation techniques in Europe, aiming to provide evidence to guide sustainable practices in AF treatment.

**Methods:**

An early environmental analysis compared pentaspline PFA (Farapulse system, Boston Scientific) with CBA using a decision-analytic model. The model simulated the patient care pathway from a hospital perspective over a 1-year time horizon, considering index and redo procedures, and complications. The environmental impact, linked to resource use, was measured in kilograms of CO_2_ equivalents (kg CO_2eq_), incorporating length of stay, intervention time, anesthetic use, and complications. Probabilistic and scenario analyses, including a comparison with RFA, were performed to assess uncertainty and robustness of the results.

**Results:**

The environmental analysis showed that PFA resulted in total emissions of 13 899 kg CO_2eq_, compared with 16 383 kg CO_2eq_ for CBA (−2483 kg CO_2eq_, −15.2%) per 100 patients. Monte Carlo simulation results confirmed these findings, showing median savings of 2409 kg CO_2eq_ (95% credible interval: 581-4312 kg). Parameters, such as anesthesia time and anesthetic drug use, were key drivers of the results. In the RFA scenario analysis, PFA yielded a saving potential of −4640 kg (−25%). In Germany, for example, with approximately 24 000 CBA procedures annually, PFA adoption was projected to reduce emissions by 509 723 kg CO_2eq_.

**Discussion:**

PFA showed potential for reducing emissions by approximately 25 kg CO_2eq_ per patient compared with CBA, driven by lower resource use. These findings aligned with studies identifying operating rooms and anesthetic drug use as major contributors to hospital emissions. A study limitation was the lack of data on catheter manufacturing and disposal.

**Conclusions:**

PFA was expected to reduce emissions compared with CBA in AF patients. Conscious medical device choices can foster more sustainable hospital practices. A full life-cycle analysis of catheters is needed to validate these findings.

## BACKGROUND

Atrial fibrillation (AF) is the most common cardiac arrythmia treated in clinical settings. It affects between 2% and 4.5% of the general population and up to 10% to 12% in populations over 80 years old.^1,2^ In Europe, paroxysmal AF is diagnosed in 25% of AF patients.^3^ Catheter ablation is a well-established approach in paroxysmal AF patients for preventing recurrence and AF manifestation. The cornerstone of this procedure involves achieving complete pulmonary vein isolation through radiofrequency ablation (RFA), cryoablation (CBA), or pulsed field ablation (PFA) techniques.^4^

While clinical efficacy and safety of ablation procedures are under continuous research, their environmental impact remains largely unexplored. Healthcare contributes approximately 5% of global greenhouse gas emissions, which is comparable to the aviation industry.^5^ One of the areas with the highest contribution to a hospital’s carbon footprint is the operating room, producing 20% to 30% of the hospital’s waste.^6^ Moreover, many anesthetic drugs are potent greenhouse gases: desflurane has a global warming potential 20 times greater than that of other anesthetics, contributing significantly to healthcare emissions. The use of these gases alone accounts for about 2% of the UK National Health Service’s (NHS) carbon footprint.^7^ Consequently, minimizing environmentally resource-intensive procedures is of growing importance for healthcare providers and purchasing departments. In anesthetic practice, the use of lower levels of sedation/anesthesia and shift toward total IV anesthesia can reduce greenhouse gas emissions substantially compared with inhalational agents, with studies indicating that using propofol instead of desflurane can lower emissions nearly 10 000-fold.^8,9^ However, the mode of anesthesia used during the procedure will also depend on geographical regulations. For example, in France or the United Kingdom (UK), it is mandatory for an anesthesiologist to be present during the procedure, whereas in other countries such as Germany, it is not required. Therefore, practices can also change based on external regulations.^10^

As environmental sustainability becomes an increasingly crucial concern, additional policies and regulations promoting sustainability within healthcare systems are being published in different countries. The NHS in the UK has set an ambitious goal to become the first national health system in the world to achieve net-zero emissions by 2040 for its direct emissions, and by 2045 for its indirect emissions. This commitment is a central part of the NHS Long Term Plan, which emphasizes sustainability as a priority for future healthcare delivery.^11^ In the Netherlands, the Dutch government is implementing the EU Green Deal by which they commit to reduce healthcare-related carbon dioxide (CO_2_) emissions by 55% by 2030, and to be climate neutral by 2050.^12,13^

PFA offers a potentially more sustainable alternative due to its improved procedural workflow by optimizing resource use around the electrophysiology (EP) lab. This is due to efficiencies around interventions resulting in reduced EP lab occupancy paired with lower anesthetic use.^14,15^ While PFA seems to be more beneficial in terms of overall costs and health-related quality of life,^16,17^ without comprehensive environmental data on ablation procedures, healthcare providers currently lack the evidence needed to make environmentally conscious treatment choices. This is despite growing clinical interest, which is also observed within the environmental goals of the European Society of Cardiology as expressed in their most recent long-term strategic plan.^18^

This research aims to address this gap in knowledge by quantifying the in-hospital environmental impact of CBA and PFA ablation techniques. This work was conducted to contribute to making holistic and informed decisions for more sustainable hospitals and to raise awareness regarding the environmental impact of resource use around technologies in paroxysmal AF treatment.

## METHODS

### Analytic Approach

To comparatively assess the environmental impact of ablation procedures and their hospital resource-related consequences, an early environmental analysis was conducted. Using Microsoft Excel, a decision-analytic model was developed comparing PFA and CBA, the two AF treatment options with the shortest procedural time. The model reflects the average paroxysmal AF in-hospital patient care pathway including index and redo procedures, as well as complications from a hospital perspective over a time horizon of 1 year. The environmental impact per intervention and comparator was expressed as kilograms of CO_2eq_. ISPOR’s good practice modeling guidelines^19^ were followed for this analysis, and reporting quality was ensured in line with the Consolidated Health Economic Evaluation Reporting Standards checklist (CHEERS 2022).^20^ The checklist is available in the **Supplementary Material**.

### Model Structure

**Figure 1.**
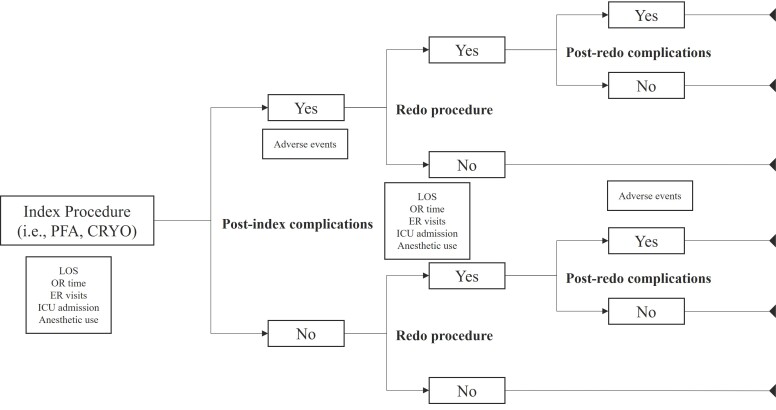
Model Structure Abbreviations: CRYO, cryoablation; ER, emergency room; ICU, intensive care unit; LOS, length of stay; OR, operating room; PFA, pulsed field ablation. Decision tree model reflecting the paroxysmal atrial fibrillation patient care pathway up to 1 cycle of redo procedures.

An early, country-agnostic, European-focused cohort model was developed in a 3-step approach to estimate the environmental impact of PFA compared with CBA for the treatment of paroxysmal AF. First, a decision tree was constructed based on a previously published cost-consequence model^15^ to outline the patient care pathway. Proportions per intervention and comparator arm were derived from the decision tree for index and redo procedures, and subsequent complications following both procedures (**Figure 1**). The model, therefore, reflects the pathway of the average patient and neither contains nor uses any individual patient-level data.

Second, the total resource use was estimated based on the proportions from the decision tree for procedures and complications. For both index procedures and redo procedures, resource use was estimated via length of hospital stay, time in the EP lab, complications requiring intensive care unit (ICU) admissions, and subsequent emergency room (ER) visits. Anesthetic time in minutes and resource use for propofol, sufentanil, and sevoflurane were estimated for both arms. Postprocedural complications considered only severe adverse events rates, including persistent phrenic nerve injury, tamponade requiring drainage (TRD), and femoral artery pseudoaneurysm. For phrenic nerve injury, outpatient visits and in-hospital check-ups were included in the model over a 1-year time horizon. Following the development of TRD, a percentage of patients received surveillance under critical care following the ablation procedure. For femoral artery pseudoaneurysm, the resource use was estimated for a proportion of patients requiring surgical repair, including additional surgical time and hospital stay.

Third, total resource use was converted into environmental units by associating each parameter with a kg CO_2eq_ (eg, kg CO_2eq_ per hospital bed or kg CO_2eq_ per hour in the EP laboratory [operating room]). Multiplying the resource use units by the environmental impact per unit provided the environmental impact per resource used (**Supplementary Table S3**). The cumulative value of the disaggregates per arm in the model provided the primary outcome of the analysis: the total environmental impact in kg CO_2eq_ per arm. The total environmental impact was first estimated and presented, based on the cohort model, for 100 patients, and then to an annual German CBA procedure level to exemplarily assess potential savings on a broader European country level.

### Model Input Data

Studies that assessed the effectiveness of CBA or PFA in terms of post-index complications, redo procedures, and post-redo complications in Europe for paroxysmal AF patients were sourced from peer-reviewed published literature following structured searches in PubMed. The literature search strategy used to retrieve the studies can be found in **Supplementary Table S1**. Due to the data available in published literature, all PFA data identified and extracted for this analysis relate to pentaspline PFA (Farapulse system, Boston Scientific).

Data for procedure-related and anesthetic model input parameters were retrieved from a secondary post-hoc analysis based on a clinical trial conducted in 6 European tertiary centers. The trial was conducted according to the principles of the Declaration of Helsinki and approved by local ethical committees (EC No.: EC-2023-208).^21^ Procedure-related data were extracted from the studies following the literature search and pooled using weighted-average methods in dependence of the number of patients involved per study. Parameters for the resource use of complications and environmental impact parameters were retrieved from peer-reviewed literature identified through targeted literature searches due to limited available data. Since data on the proportion of patients with TRD requiring critical care were not available, a conservative assumption based on the input from the clinical authors of the present manuscript was used instead.

### Model Uncertainty

Uncertainty of the model was addressed using sensitivity analyses. To assess uncertainty around the deterministic base case results, a 2000-iteration Monte Carlo simulation was run, varying chosen input parameters at the same time based on attributed distributions (**Table 1**). Beta and normal distributions were used for proportions. Normal distributions were assigned to continuous variables assuming that the data applied in the cohort model represents the average patient under the central limit theorem. Standard deviations of model parameters to estimate the lower and upper bounds of the distributions were either retrieved from the literature or calculated based on an assumed variance of 10%. Results of this probabilistic sensitivity analysis were presented as median differences between intervention and comparator arms and uncertainty expressed as Bayesian 95% credible intervals (CrI).^22^ A CrI not crossing zero means that the result (total savings in CO_2eq_) can be interpreted as statistically significant. The 2000 simulation results were visualized using a bar chart. To estimate the impact of different parameters on the results, each model parameter was varied deterministically by ±20% in a one-way sensitivity analysis to estimate the sensitivity of the overall results to certain parameters. The results were recorded and plotted in a tornado diagram.

**Table 1.** Model Input Parameters

**Parameter**	**PFA**	**CBA**	**Unit**	**Distribution**	**Reference**
Length of stay (LOS)					
General ward/cardiology	2.40	2.41	Days	Normal	van de Kar et al (2024),^15^ Chierchia et al (2024)^21^^a^
ICU	1.00	2.00	Days	Normal	Chierchia et al (2024)^21^^a^
Procedure					
Total procedure time	65.77	77.22	Minutes	Normal	Chierchia et al (2024),^21^^a^ van de Kar et al (2024),^15^ Maurhofer et al (2024),^23^ Della Rocca et al (2023),^24^ Reddy et al (2023)^25^
Additional resource use					
Redo procedures	13.37	16.47	%	Beta	Maurhofer et al (2024),^23^ Della Rocca et al (2023)^24^
ER visits	1.49	0.74	%	Beta	Chierchia et al (2024)^21^^a^
ICU admissions	0.75	1.48	%	Beta	Chierchia et al (2024)^21^^a^
Anesthetics					
Anesthesia time	64.91	84.76	Minutes	Normal	Chierchia et al (2024)^21^^a^
Propofol use	99.00	99.00	%	Beta	Chierchia et al (2024)^21^^a^
Sufentanil use	70.15	20.00	%	Beta	Chierchia et al (2024)^21^^a^
Sevoflurane use	19.40	66.67	%	Beta	Chierchia et al (2024)^21^^a^
Adverse events					
Permanent PNI	0.00	0.63	%	Beta	Chierchia et al (2024),^21^^a^ Della Rocca et al (2023),^24^ Reddy et al (2023)^25^
TRD	0.24	0.29	%	Beta	Chierchia et al (2024),^21^^a^ Maurhofer et al (2024),^23^ Della Rocca et al (2023),^24^ Reddy et al (2023)^25^
FAP	0.75	0.74	%	Beta	Chierchia et al (2024)^21^^a^
Interventions following adverse events					
PNI patients with outpatient visits	56.90		%	Beta	Calculated from Tokuda et al (2021)^26^
No. of outpatient visits of PNI patients	4			Normal	Tokuda et al (2021)^26^
TRD patients requiring critical care	50		%	Beta	Assumption
TRD patients LOS in ICU	1.99		Days	Normal	Stremmel et al (2021)^27^
FAP patients requiring surgical repair	1.68		%	Beta	Savolainen et al (2012)^28^
FAP surgical repair time	69.70		Minutes	Normal	Ibrahim et al (2017)^29^
FAP patient LOS for surgical repair	11		Days	Normal	Savolainen et al (2012)^28^
CO_2eq_/resource use					
CO_2eq_/bed day	38		kg	Normal	Penny et al (2015)^30^
CO_2eq_/ICU day	90		kg	Normal	Penny et al (2015)^30^
CO_2eq_/hour in operating room (excluding anesthetics)	24.06		kg	Normal	Whiting et al (2020)^31^^b^
CO_2eq_/ER visit	14		kg	Normal	Penny et al (2015)^30^
CO_2eq_/outpatient visit	1.1		kg	Normal	Penny et al (2015)^30^
CO_2eq_/hour of propofol use^a^	0.01		kg	Normal	Kalmar et al (2023)^32^
CO_2eq_/hour of sufentanil use^a^	0.01		kg	Normal	Pearson et al (2022)^33^
CO_2eq_/hour of sevoflurane use^a^	0.25		kg	Normal	Kalmar et al (2023)^32^

Abbreviations: CBA, cryoablation; ER, emergency room; FAP, femoral artery pseudoaneurysm; ICU, intensive care unit; LOS, length of stay; PFA, pulsed field ablation; PNI, phrenic nerve injury; TRD, tamponade requiring drainage.

^a^For more information, contact D.R., co-investigator of the PERFECT-PAF trial.

^b^Retrieved from Whiting et al and normalized to Penny et al using CO_2eq_ unit values per hospital day as a proxy.

Four scenario analyses were conducted to allow for validation and interpretation of the results. A first scenario analysis was conducted varying the fresh gas flow of sevoflurane, which was conservatively assumed to be 0.3 L/min. Firstly, in line with a previously published paper, sevoflurane gas flow was increased to 2 L/min and 6 L/min.^32^ Second, in an additional attempt to model an approach using only total IV anesthesia (only assessing propofol and sufentanil), no sevoflurane fresh gas flow (0 L/min) was assumed across both arms. A second scenario analysis included the articles from the structured literature search that reported the effectiveness of CBA for mixed populations of paroxysmal and persistent patients. A third scenario analysis was designed approximating the environmental impact of RFA using resource use data inputs from a recently published paper retrieved from the structured literature review.^15^ For this scenario, the anesthetic drug use and complications were kept constant when comparing PFA and RFA. Since the published paper did not contain all resource use parameters used in the analysis, the following parameters were used for RFA while the rest of the model input parameters were kept constant: total procedure time (140.2 min), redo procedure rates (19.5%), anesthesia time (110.1 min).^15^ A fourth scenario analysis was designed to model the impact of carbon intensity of electricity, which differs by European country. Based on CO_2eq_ emissions, expressed as gram per kilowatt hour (g/kWh), on the UK grid and relative multiplicators derived from data on the 27 countries of the European Union,^34,35^ we approximated the impact of a varying the energy mix on the CO_2eq_ unit parameters for resource use and subsequently on the base case results.

## RESULTS

### Structured Literature Review

The structured literature review retrieved a total of 21 articles, of which 9 were included for full-text screening (see **Supplementary Figure S1** for PRISMA flow diagram). After full-text screening, 8 articles were included for data extraction and pooling. Articles were excluded for either being set outside Europe (1 article), only consisting of a study protocol (3), not listing outcomes of interest for our analysis (4), not listing both intervention (PFA) and comparator (CBA) (4), or not yielding primary data (1). The pooled results on procedural data, the complication-related, and environmental model input parameters are presented in **Table 1**.

### Deterministic Results

The early model, assessing the environmental impact of ablation procedures and its in-hospital resource use comparing PFA and CBA, resulted in total CO_2eq_ of 139 kg for PFA and 164 kg for CBA (-15.2%). For a cohort of 100 paroxysmal AF patients, reflecting the annual patient volume in a small- to medium-sized hospital, the model resulted in savings of 2483 kg of CO_2eq_. **Table 2** shows a breakdown of the results by index and redo procedure as well as by procedure-related complications. Exploring the individual resource parameter contribution to savings, anesthetics use contributed 52%, LOS-related CO_2eq_ 14%, EP lab time 25%, ER visits and ICU admissions 9%, and complications 0.25%. When considering the yearly number of CBA procedures conducted in Germany (~24 000),^36^ and under an assumption of an average redo procedure rate of 14.9% across the PFA and CBA arms, the model results in CO_2eq_ emissions of 2 852 765 kg (PFA) and 3 362 488 kg (CBA). The subsequent CO_2eq_ savings through PFA use in AF ablation procedures add up to 509 723 kg.

**Table 2.** Base Case Results: Environmental Impact per 100 Paroxysmal Atrial Fibrillation Patients

**Event**	**PFA (CO_2eq_ kg)**	**CBA (CO_2eq_ kg)**	**Difference (CO_2eq_ kg)**	**CO_2eq_ kg Contribution (%)^a^**
Index procedure	12 242	14 044	−1802	72.5
Post-index complications	17.8	22	−4.2	0.2
Redo procedure	1637	2313	−676	27.2
Post-redo complications	2.4	3.6	−1.2	0.05
Total	**13 899**	**16 383**	**−2483**	**100**

Abbreviations: CBA, cryoablation; PFA, pulsed field ablation.

^a^Percentage of contribution to overall savings.

### Sensitivity Analyses

The deterministic results of the base case were confirmed by the results of the 2000-iteration Monte Carlo simulation. The analysis resulted in median CO_2eq_ savings of 2409 kg when comparing PFA to CBA, with a 95% CrI ranging from 581 kg to 4312 kg. This translates to a median percentage reduction of 14.8% (95 CrI, 3.8%-24.3%). The environmental impact of PFA was lower in 99.8% of iterations. **Supplementary Figure S2** plots the 2000 results of the Monte Carlo simulation.

The one-way sensitivity analysis revealed that the results were mainly driven by anesthesia time, the use and environmental impact of sevoflurane, and redo procedure rates when varying the input parameters by ±20%. Additional parameters with a modest impact on the results were the emissions per bed day, the length of stay, and procedure-related parameters such as the environmental impact of, and the time in the EP lab. The two environmental parameters with the biggest impact on the results were anesthesia time and the CO_2eq_ impact of sevoflurane use. The variation of the CO_2eq_ impact for both parameters by ±20% increased/decreased the total environmental impact by 10.5% and 10.4%, respectively. The results of the one-way sensitivity analysis confirmed the minor impact of the parameter assumptions on the total results (±2.8%), which included the percentage of patients with TRD requiring critical care (see **Supplementary Figure S3** for tornado plot).

In the first scenario analysis, increasing the fresh gas flow of sevoflurane from 0.25 L/min to 2 L/min led to a change in the CO_2eq_ difference between PFA and CBA of +465% (11,547 kg) compared with the base case (2483 kg). Increasing the fresh gas flow to 6 L/min led to 32,265 kg in environmental savings, which translates to a 1299% increase over the base case. Assuming no sevoflurane gas use across both arms lowered the overall environmental savings by 48% (by -1189 kg) compared with the base case. This scenario analysis is in line with the results of the one-way sensitivity analysis, where the use of sevoflurane in both the comparator and intervention group as well as the environmental impact of the gas were found to be the second and third most impactful parameters in terms of the total model results.

In the second scenario analysis, studies reporting resource use for mixed populations (paroxysmal and persistent AF patients) were included (**Supplementary Table S2**). Compared with the paroxysmal-only pooled data, using mixed population data led to slightly higher overall CO_2eq_ savings per 100 patients (+188 kg, +7.6%). Noticeably, index procedures contributed 78% to the savings (with 72% in the base case), which is likely driven by reduced procedure times (relative reduction rates in this scenario and the base case respectively: 20.6% vs 14.8%). Moreover, post-index procedure complications were higher for PFA than for CBA; however, they had a marginal contribution to the overall results (**Table 3**).

**Table 3.** Scenario Analysis For Mixed Population Studies: Results per 100 Patients

**Event**	**PFA (CO_2eq_ kg)**	**CBA (CO_2eq_ kg)**	**Difference (CO_2eq_ kg)**	**CO_2eq_ kg Contribution (%)^a^**
Index procedure	12 454	14 538	−2084	78.0
Post-index complications	24.5	20.2	4.3	−0.2
Redo procedure	1380	1972	c592	22.2
Post-redo complications	2.7	2.7	0.0	0.0
**Total**	**13 861**	**16 533**	**−2671**	**100**

Abbreviations: CBA, cryoablation; PFA, pulsed field ablation.

^a^Percentage of contribution to overall savings.

The third scenario analysis, comparing PFA and CBA to an approximated RFA arm, resulted in environmental savings for both base case interventions (PFA, CBA). Compared with PFA, RFA had an increased environmental impact of 4640 kg CO_2eq_ per 100 patients. Compared with the base case savings of PFA against CBA, this comparison amplified the savings by 86%. This result was mainly driven by higher procedure times (RFA, 140.2 min vs PFA, 65.77 min), anesthesia times (RFA, 110.1 min vs PFA, 64.91 min), and redo procedure rates (RFA, 19.5% vs PFA, 13.4%). In this scenario, 80% of the environmental impact savings were attributed to the time in the EP lab. **Table 4** visualizes the result comparison across all three arms. The results of the fourth scenario analysis, varying the impact of the energy grid, can be found in **Supplementary Table S4**.

**Table 4.** RFA Scenario: Environmental Impact per 100 Paroxysmal AF Patients

**Event**	**PFA (CO_2eq_ kg)**	**CBA (CO_2eq_ kg)**	**RFA (CO_2eq_ kg)**	**Difference PFA/RFA (CO_2eq_ kg)**
Index procedure	12 242	14 044	15 497	−3255
Redo procedure	1637	2313	3022	−1385
**Total**	**13 879**	**16 357**	**18 519**	**−4640**

Abbreviations: CBA, cryoablation; PFA, pulsed field ablation.

## DISCUSSION

Our study provides an early comparative environmental model of two commonly employed cardiac ablation techniques, pentaspline PFA and CBA, for the treatment of AF. The present study is, to our knowledge, the first to assess the carbon footprint associated with the ablation procedures in hospital settings, contributing to holistic value-based healthcare decision making by considering resources used during index procedures, its associated complications, and subsequent redo procedures.^37,38^ The results of our analysis indicate that PFA might offer an approach to lower carbon emissions by 25 kg CO_2eq_ (95% CrI, 6-43) per patient compared with CBA. This impact is primarily driven by reduced redo rates as well as procedure and anesthetic times associated with PFA. This, in turn, can improve organizational efficiency and allow for more streamlined processes through decreasing EP laboratory time utilization.

While resource efficiencies might enable higher procedural throughput and increase the overall emissions from a hospital perspective, the benefit will be tangible on a country level, as seen in the German population scenario. On a population level, overall CO_2eq_ emissions in Germany might be decreased by 509 723 kg when considering the base case results. Taking into consideration the conservative lower bound of the 95% CrI (savings per procedure, 6 kg CO_2eq_) and an average emission of a newly registered vehicle in the European Union (106.4 g CO_2eq_/km),^39^ the annual savings comparing PFA and CBA translate to a driving distance of 1 288 886 km. This is equivalent to one car driving all existing roads in Germany roughly 1.5 times.^40^

Overall, the base case results highlight the substantial environmental impact of healthcare interventions, particularly those involving resource-intensive procedures such as operating room and sedation procedures.^31,32^ The procedural impact results are underscored by our second scenario analysis, where the environmental impact of resource use in PFA and CBA in mixed populations (paroxysmal and persistent AF) was examined. The environmental savings per 100 patients increased by 188 kg CO_2eq_, driven by procedural efficacies and lower redo rates. Further research should be undertaken to explore these savings in persistent AF patients.

The findings are in line with those of other environmental impact resource use studies. A carbon footprint study found operating theaters to be the dominant contributor to both emissions and waste generation in the hospital settings, producing 3 to 6 times more greenhouse gases than all other areas in the hospital.^41^ Another recent study assessing the carbon footprint of AF catheter ablation with RFA or CBA reported the anesthesia workstation to contribute to 24.6% of the carbon emissions related to the ablation procedure.^42^ This is in line with the findings of our scenario analysis, where a 20% increase in fresh gas flow of sevoflurane increased the total emissions in the CBA arm by up to 10.4%. Consequently, when anesthetic drugs and gases are utilized, shorter anesthesia and procedure times can contribute to environmental savings. The magnitude of savings can depend on factors such as the choice of the drug and the amount used, as seen in the scenario analysis where the fresh gas flow of sevoflurane was varied to approximate heterogeneous anesthetic practices. It should be noticed that these practices can differ between countries and hospital types due to varying workflows, regulations and protocols. Additionally, the third scenario analysis, approximating the environmental impact of RFA, showed the impact of prolonged EP lab and anesthetic times. Both parameters, which are higher for RFA, drive the results contributing to increased savings of 25% (−4640 kg) when comparing PFA and RFA. The indication of this scenario analysis showcases the importance of efficient practice choices and postulates further research in a full comparison between PFA and RFA upon data availability.

When interpreting the results in comparison with other published data, a recent study showed the carbon footprint of AF ablation with CBA and RFA to be on average 76.9 kg of CO_2eq_, assessing the EP laboratory procedure and catheter material environmental impact.^42^ In our work, preoperative and postoperative phases of hospitalization were not considered (ie, transportation to the hospital, postintervention length of stay). While manufacturing, catheter material packaging, and transport were not included in our analysis, it would be valuable to consider it in a full life-cycle analysis (LCA). One strength of our analysis is that resource use and consequences of the interventions beyond the ablation procedure itself were considered.

Countries like the UK and the Netherlands have taken significant steps to promote sustainable healthcare. The UK’s NHS has set ambitious targets to achieve net-zero emissions by 2050, while the Netherlands is implementing the EU Green Deal, which aims to reduce by 30% the carbon emissions from healthcare buildings and energy consumption by 2026.^11,12^ These policies highlight the growing recognition of the environmental impact of health care and the need for comprehensive strategies.

To further minimize the environmental impact of catheter ablation, additional strategies can be considered beyond hospital resource use. A recent physician survey conducted by the EHRA/LIRYC Scientific Committee reported that education on best practices, recommendations from companies on post-procedure use, financial incentives, and take-back services from companies were some of the potential sustainability solutions that could be implemented.^43^ Additionally, another strategy to decrease the ablation procedure carbon footprint would be developing tools such as mobile applications to reduce the number of hospital visits pre- and post-intervention needed for patient education and follow-up.^44,45^ Streamlining the orders and logistical operations to ensure efficient delivery of catheter ablation products could contribute to lower the carbon emissions related to AF ablation procedures, since transport contributes to 10.6% of the AF procedure carbon emissions.^42^ From a population perspective, the energy mix used to provide AF procedures plays a crucial role, as highlighted in our fourth scenario analysis. The total savings in CO_2eq_ kg differed from the base case by −13.8 kg in a low-carbon (Sweden) to 69.7 kg in a high-carbon (Poland) setting, which translates into a deviation from our base case results of −44.6% and +180.7%, respectively.

The present study is not free of limitations. The main constraint is the limited, country-specific data populating the model. The environmental impact parameters were primarily derived from the UK; however, some of the other model parameters were derived from studies conducted in other European countries. As clinical practice might vary (eg, in anesthesia agents used or redo interventions chosen), the use of such proxy values adds uncertainty to the interpretation of model outcomes. Additionally, even though publication of environmental impact data has been increasing in recent years, it is still scarce for specific procedures and resource uses. This leads to the inability to incorporate all relevant parameters to the model. The anesthetic drug use for PFA and CBA was extracted from a single publication, which may limit the generalizability of the environmental impact associated with anesthetic drug use, especially due to anesthetic practices varying widely between countries. Redo procedures were modeled to be executed with the same intervention (PFA, CBA, RFA) as the index procedure. This might not apply to all clinical practices and ablation devices. The PFA data incorporated into this model were retrieved from studies using the Farapulse system. Generalization of the results across other PFA devices such as spherical or variable loop catheter might, therefore, be limited due to differences in device specifications such as the number of electrodes or the circumferential of the systems. Further research to compare different PFA techniques is warranted. Moreover, the model did not account for the entire lifecycle of medical devices, including production, disposal, or patient and caregiver transportation, which impacts the overall environmental footprint of AF catheter ablation. Lastly, the present study assesses the environmental parameter of CO_2eq_, which is not necessarily a proxy for more sustainable procedures. Performing a full LCA was not in the scope of this study; therefore, other environmental impacts such as freshwater usage or human toxicity were not assessed.

To address some of the limitations and advance the field of sustainable healthcare, future research should prioritize collecting detailed primary data on resource utilization and environmental metrics for various medical procedures. This should also include studies to estimate the waste generation of procedures and the impact of resource efficiencies such as avoided reinterventions. At the same time, awareness regarding challenges concerning the switch and uptake of environmentally efficient practices should be fostered.^46^ Once additional data are available, further analyses can be conducted to assess the environmental impact of catheter ablation in paroxysmal AF patients in different country settings, including other resource use outcomes and manufacturing-related carbon emissions.

## CONCLUSION

The results of this early environmental analysis show that pentaspline PFA is projected to reduce emissions compared to CBA during cardiac ablation in paroxysmal AF patients. Although these results should be interpreted with caution due to the limited data in the field, they highlight the significant impact of ablation procedures in general. While this initial analysis addressed only the in-hospital resource use within the full product life cycle of an AF ablation catheter, it shows that consideration of the wider impacts of medical device use may reduce the environmental implications of procedures and resource use in hospitals. A comprehensive LCA for PFA and CBA catheters is missing and should be addressed with future research undertakings. Overall, with the urge for more awareness around environmental impact within European society and reflected in published policies, more data are needed to routinely assess the environmental impact of interventions alongside health-economic evaluations.

## Supplementary Material

Online Supplementary Material

## Data Availability

For access to the analytical source code of the model, please contact the corresponding author.
