# Supplementary material for: Early Assessment of the Environmental Impact of Pentaspline Pulsed Field Ablation and Cryoablation in the Treatment of Paroxysmal Atrial Fibrillation
Source: J Health Econ Outcomes Res. 2025 Dec 15;12(2):237–44. doi: 10.36469/001c.151216 (PMC12710608; doi:10.36469/001c.151216)
Supplement: Online Supplementary Material [file jheor_2025_12_2_151216_322183.pdf]

Journal of Health Economics and Outcomes Research

Online Supplementary Material

Early Assessment of the Environmental Impact of Pentaspline Pulsed Field Ablation and Cryoablation in the Treatment of Paroxysmal Atrial Fibrillation. *JHEOR*. 2025;12(2):237-244. doi:10.36469/jheor.2025.151216

Table S1: Structured Searches in PubMed to Identify Relevant Modeling Data

Table S2: Mixed Population Input Parameters for Scenario Analysis

Figure S1: Prisma Flow Diagram for the Structured Literature Review

Figure S2: Results of the Probabilistic Sensitivity Analysis

Figure S3: One-Way Sensitivity Analysis Results

Equation: Formula for Calculation of the Environmental Impact per Decision Node

Table S3: Energy Grid Scenario Analysis

CHEERS Checklist

This supplementary material has been provided by the authors to give readers additional information about their work.

This is an open-access article distributed under the terms of the Creative Commons Attribution 4.0 International License (CCBY-4.0). View this license's legal deed at http://creativecommons.org/licenses/by/4.0 and legal code at http://creativecommons.org/licenses/by/4.0/legalcode for more information.

Figure S1 visualizes the decision tree and the resource use inclusion at each decision node (index and redo procedures as well as adverse events). Model inputs can be retrieved from **Table S1**. CO_{2eq} unit values were not adjusted for hospital-specific infrastructure for this early analysis due to a lack of data granularity.

Table S1. Structured Searches in PubMed to Identify Relevant Modeling Data

Index	Aim	Search String	Hits
1	PFA	(farapulse[tiab] OR "pulsed field ablation"[tiab] OR "pulsed-field ablation"[tiab] OR "pulse-field ablation"[tiab] OR "pulse-field ablation"[tiab])	657
2	CRYO	(cryoballoon[tiab] OR cryo[tiab] OR "cryo balloon"[tiab])	27 001
3	AF	((atrial[tiab] OR auricular[tiab] OR cardiac[tiab] OR heart[tiab]) AND (fibrillation[tiab] OR fibrillations[tiab] OR arrhythmia[tiab]))	152 532
4	Study types	(randomized[tiab] OR randomised[tiab] OR controled[tiab] OR controlled[tiab] OR blinded[tiab] OR double-blind[tiab] OR randomly[tiab] OR double-blinded[tiab] OR "real world"[tiab] OR "real-world"[tiab] OR registry[tiab] OR registry[tiab] OR "Propensity score matching"[tiab])	2 145 411
5	Non-clinical data	"ex vivo"[tw] OR "ex-vivo"[tw] OR cadaver[tw] OR cadaveric[tw] OR "deceased donor"[tw]	185 508
6	Excluded study types	Address[pt] OR Autobiography[pt] OR Biography[pt] OR Bibliography[pt] OR "Case Reports"[pt] OR "Clinical Conference"[pt] OR "Clinical Trial, Veterinary"[pt] OR "Collected Work"[pt] OR Comment[pt] OR Congress[pt] OR "Consensus Development Conference"[pt] OR "Consensus Development Conference, NIH"[pt] OR Dictionary[pt] OR Directory[pt] OR "Duplicate Publication"[pt] OR Editorial[pt] OR "Expression of Concern"[pt] OR Festschrift[pt] OR "Historical article"[pt] OR "Interactive tutorial"[pt] OR Interview[pt] OR "Introductory Journal Article"[pt] OR Lecture[pt] OR "Legal Case"[pt] OR Legislation[pt] OR Letter[pt] OR News[pt] OR "Newspaper Article"[pt] OR "Observational Study, Veterinary"[pt] OR "Patient Education Handout"[pt] OR "Periodical Index"[pt] OR "Personal Narrative"[pt] OR Portrait[pt] OR "Published Erratum"[pt] OR "Randomized Controlled Trial, Veterinary"[pt] OR "Retracted Publication"[pt] OR "Retraction of Publication"[pt] OR "Technical Report"[pt] OR "Twin Study"[pt] OR "Video-Audio Media"[pt] OR Webcast[pt] OR "Systematic Review"[pt] OR Review[pt]	8 898 419
7	All exclusions	#5 OR #6	9 060 260
8	All relevant studies	#1 AND #2 AND #3 AND #4 NOT #7	21

Performed 28 January 2025.

Table S2. Mixed Population Input Parameters for Scenario Analysis

Parameter	PFA	CBA	Unit	Distribution	Reference
Length of stay (LOS)					
General ward/cardiology	2.40	2.41	Days	Normal	van de Kar et al (2024),[15] Chierchia et al (2024)[21a]
ICU	1.00	2.00	Days	Normal	Chierchia et al (2024)[21a]
Procedure					
Total procedure time	69.12	87.11	Minutes	Normal	Chierchia et al (2024),[21a] van de Kar et al (2024),[15] Maurhofer et al (2024),[26] Della Rocca et al (2023),[27] Reddy et al (2023),[28] Calvert et al (2024),[47] Chaumont et al (2024),[48] Blockhaus et al (2023),[49] van de Kar et al (2024)[50]
Additional resource use					
Redo procedures	11.08	13.57	%	Beta	Della Rocca et al (2023),[27] Calvert et al (2024),[47] Chaumont et al (2024),[48] van de Kar et al (2024),[50] Maurhofer et al (2024)[26]
ER visits	1.49	0.74	%	Beta	Chierchia et al (2024)[21a]
ICU admissions	0.75	1.48	%	Beta	Chierchia et al (2024)[21a]
Anesthetics					
Anesthesia time	64.91	84.76	Minutes	Normal	Chierchia et al (2024)[21a]
Propofol use	99.00	99.00	%	Beta	Chierchia et al (2024)[21a]
Sufentanil use	70.15	20.00	%	Beta	Chierchia et al (2024)[21a]
Sevoflurane use	19.40	66.67	%	Beta	Chierchia et al (2024)[21a]
Adverse events					
Permanent PNI	0.01	0.59	%	Beta	Chierchia et al (2024),[21a],Della Rocca et al (2023),[27] Reddy et al (2023),[28] Calvert et al (2024),[47] Chaumont et al (2024),[48] Blockhaus et al (2023),[49], van de Kar et al (2024)[50]
TRD	0.37	0.25	%	Beta	Chierchia et al (2024),[21a] Maurhofer et al (2024),[26] Della Rocca et al (2023),[27] Reddy et al (2023),[28] Calvert et al (2024),[47] Chaumont et al (2024),[48] Blockhaus et al (2023),[49] van de Kar et al (2024)[50]
FAP	0.75	0.74	%	Beta	Chierchia et al (2024)[21a]

Abbreviations: CBA, cryoablation; ER, emergency room; FAP, femoral artery pseudoaneurysm; ICU, intensive care unit; LOS, length of stay; PFA, pulsed field ablation; PNI, phrenic nerve injury; TRD, tamponade requiring drainage.
[a]For further information, contact D.R., co-investigator of the PERFECT-PAF trial.

Figure S1. Prisma Flow Diagram for the Structured Literature Review

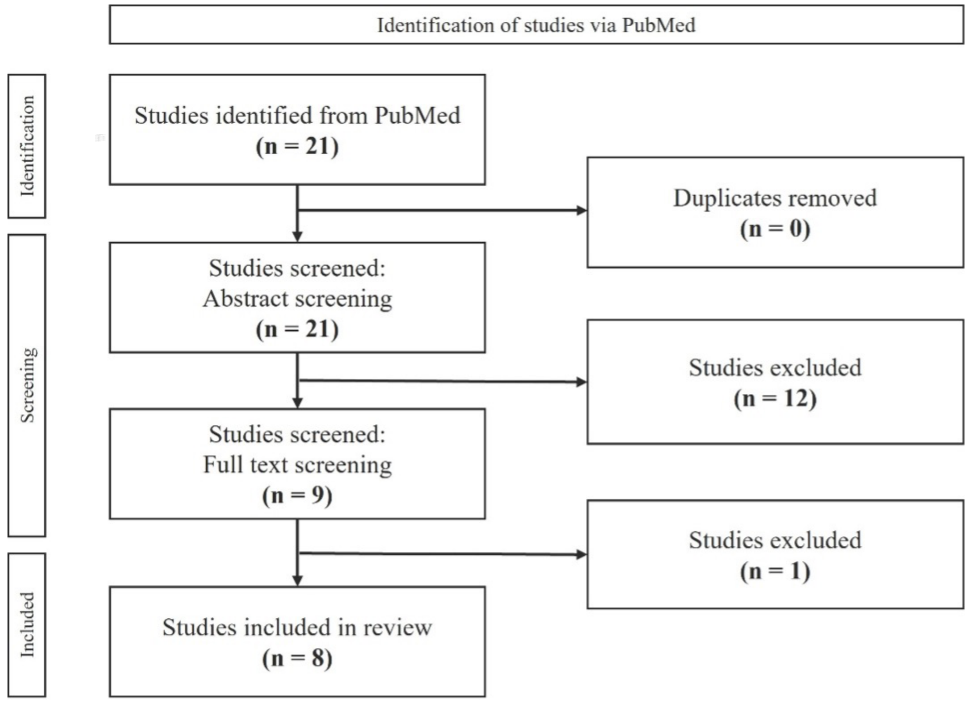

Figure S2. Results of the Probabilistic Sensitivity Analysis

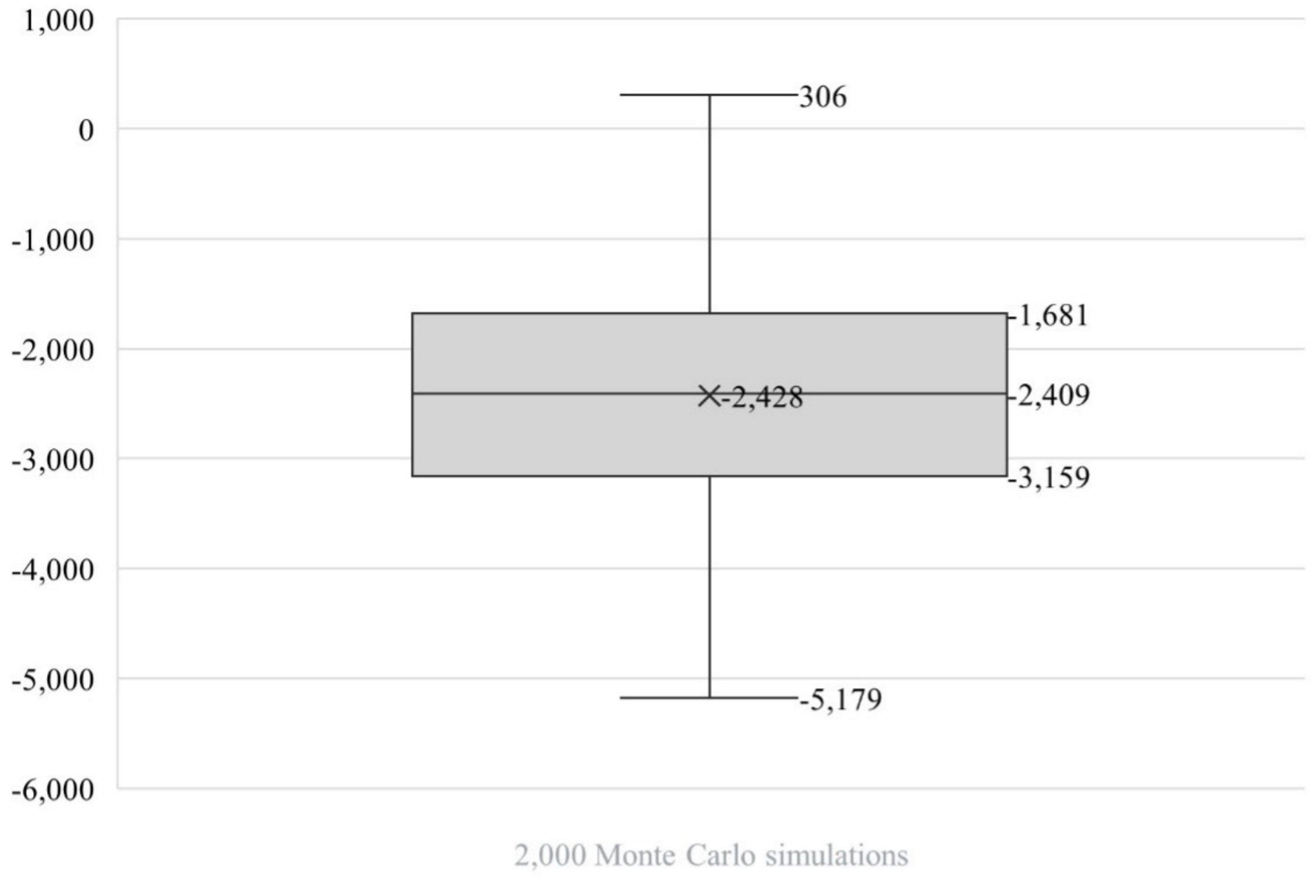

Note: Box plot presenting the results (per 100 paroxysmal atrial fibrillation patients) of the probabilistic sensitivity analysis: environmental impact difference between PFA and cryoablation (in kg CO_{2eq}). The box indicates the interquartile range (–1681 to –3159), the solid line in the box is the median value (–2409), and X denotes the mean value, 2428.

Figure S3. One-Way Sensitivity Analysis Results

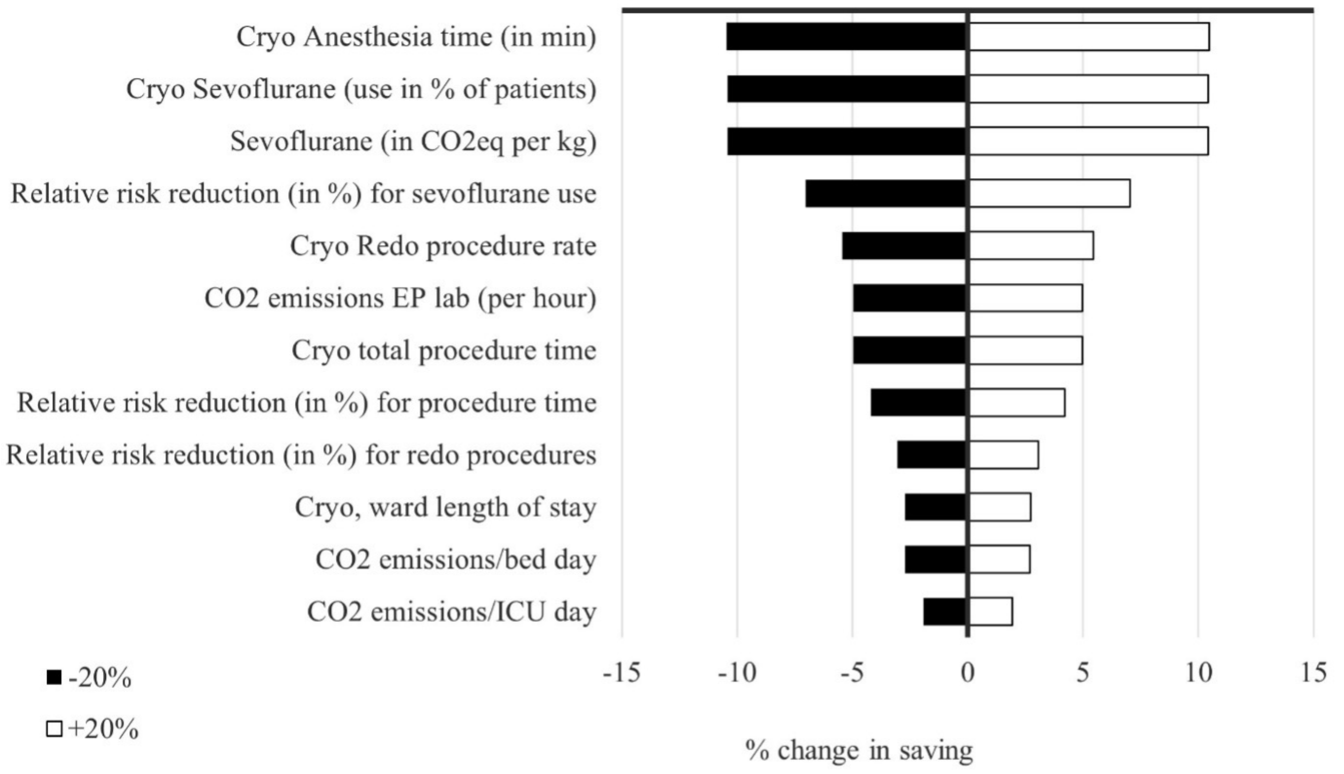

Abbreviations: Cryo, cryoablation; EP, electrophysiology; LOS, length of stay, OR time, operating time.
Note: The tornado plot visualizes the effects of deterministically varying single parameters (left hand side) by ±20%, capturing the percentage change of the total environmental difference between PFA and CBA. In the model, the difference between CBA and PFA was captured using relative risk reductions, based on the pooled analysis. Therefore, the baseline parameters are captured for the CBA arm only, while the PFA values were captured using the relative risk reduction (e.g., relative risk reduction in sevoflurane).

Equation: Formula for Calculation of the Environmental Impact per Decision Node

Decision Tree Proportion × Resource Use × Unit in CO_{2eq}

Table S3. Energy Grid Scenario Analysis

Country	Emissions ($g CO_{2eq}$ per kWh)	Relative Factor	Total Savings ($kg CO_{2eq}$)	Deviation from Base Case (%)
UK	124	1.00	-24.83	0.0
Sweden	8	0.06	-13.76	-44.6
Finland	33	0.27	-16.15	-35.0
Luxembourg	41	0.33	-16.91	-31.9
France	48	0.39	-17.58	-29.2
Austria	59	0.48	-18.63	-25.0
Latvia	68	0.55	-19.49	-21.5
Denmark	76	0.61	-20.25	-18.5
Lithuania	78	0.63	-20.44	-17.7
Slovakia	83	0.67	-20.92	-15.8
Portugal	115	0.93	-23.98	-3.5
Croatia	139	1.12	-26.27	5.8
Spain	140	1.13	-26.36	6.2
Hungary	142	1.15	-26.55	6.9
Belgium	150	1.21	-27.32	10.0
Slovenia	166	1.34	-28.84	16.1
EU-27 average	207	1.67	-32.76	31.9
Romania	226	1.82	-34.57	39.2
Italy	250	2.02	-36.86	48.4
Ireland	255	2.06	-37.34	50.4
Netherlands	256	2.06	-37.44	50.7
Bulgaria	293	2.36	-40.97	65.0
Germany	320	2.58	-43.55	75.3
Malta	341	2.75	-45.55	83.4
Greece	343	2.77	-45.74	84.2
Czechia	361	2.91	-47.46	91.1
Estonia	538	4.34	-64.36	159.2
Cyprus	575	4.64	-67.89	173.4
Poland	594	4.79	-69.71	180.7

CHEERS Checklist

Topic	No.	Item	Location Where Item Is Reported
Title	1	Identify the study as an economic evaluation and specify the interventions being compared.	NA
Abstract	2	Provide a structured summary that highlights context, key methods, results, and alternative analyses.	Abstract (pages 0/1)
Introduction			
Background and objectives	3	Give the context for the study, the study question, and its practical relevance for decision making in policy or practice.	Pages 2/3 (Background)
Methods			
Health economic analysis plan	4	Indicate whether a health economic analysis plan was developed and where available.	Not available
Study population	5	Describe characteristics of the study population (such as age range, demographics, socioeconomic, or clinical characteristics).	Pages 5/6 (Methods)
Setting and location	6	Provide relevant contextual information that may influence findings.	Page 4/5 (Methods)
Comparators	7	Describe the interventions or strategies being compared and why chosen.	Page 4/5 (Methods)
Perspective	8	State the perspective(s) adopted by the study and why chosen.	Pages 4/5 (Methods)
Time horizon	9	State the time horizon for the study and why appropriate.	Page 4 (Methods)
Discount rate	10	Report the discount rate(s) and reason chosen.	NA
Selection of outcomes	11	Describe what outcomes were used as the measure(s) of benefit(s) and harm(s).	Pages 5/6 (Methods)
Measurement of outcomes	12	Describe how outcomes used to capture benefit(s) and harm(s) were measured.	NA
Valuation of outcomes	13	Describe the population and methods used to measure and value outcomes.	NA
Measurement and valuation of resources and costs	14	Describe how costs were valued.	NA
Currency, price date, and conversion	15	Report the dates of the estimated resource quantities and unit costs, plus the currency and year of conversion.	NA
Rationale and description of model	16	If modelling is used, describe in detail and why used. Report if the model is publicly available and where it can be accessed.	Pages 4/5/6/7/8 (Methods)
Analytics and assumptions	17	Describe any methods for analysing or statistically transforming data, any extrapolation methods, and approaches for validating any model used.	Page 6 (Methods)
Characterizing heterogeneity	18	Describe any methods used for estimating how the results of the study vary for subgroups.	NA
Characterizing distributional effects	19	Describe how impacts are distributed across different individuals or adjustments made to reflect priority populations.	NA
Characterizing uncertainty	20	Describe methods to characterise any sources of uncertainty in the analysis.	Pages 7/8 (Methods)
Approach to engagement with patients and others affected by the study	21	Describe any approaches to engage patients or service recipients, the general public, communities, or stakeholders (such as clinicians or payers) in the design of the study.	NA

CHEERS Checklist

Topic	No.	Item	Location Where Item Is Reported
Results			
Study parameters	22	Report all analytic inputs (such as values, ranges, references) including uncertainty or distributional assumptions.	Table 1 (Results)
Summary of main results	23	Report the mean values for the main categories of costs and outcomes of interest and summarise them in the most appropriate overall measure.	Pages 8/9 (Results)
Effect of uncertainty	24	Describe how uncertainty about analytic judgments, inputs, or projections affect findings. Report the effect of choice of discount rate and time horizon, if applicable.	Pages 9/10/11 (Results)
Effect of engagement with patients and others affected by the study	25	Report on any difference patient/service recipient, general public, community, or stakeholder involvement made to the approach or findings of the study	NA
Discussion			
Study findings, limitations, generalizability, and current knowledge	26	Report key findings, limitations, ethical or equity considerations not captured, and how these could affect patients, policy, or practice.	Pages 11/12/13/14/15 (Discussion)
Other relevant information			
Source of funding	27	Describe how the study was funded and any role of the funder in the identification, design, conduct, and reporting of the analysis	Reported in disclosure
Conflicts of interest	28	Report authors conflicts of interest according to journal or International Committee of Medical Journal Editors requirements.	Reported in disclosure

Abbreviation: NA, not applicable.
